# A Real-Time Subway Driver Action Sensoring and Detection Based on Lightweight ShuffleNetV2 Network

**DOI:** 10.3390/s23239503

**Published:** 2023-11-29

**Authors:** Xing Shen, Xiukun Wei

**Affiliations:** 1 School of Traffic and Transportation, Beijing Jiaotong University, Beijing 100044, China; 2 State Key Laboratory of Advanced Rail Autonomous Operation, Beijing Jiaotong University, Beijing 100044, China

**Keywords:** action recognition, deep learning, driver detection, railway, action sensoring and detection

## Abstract

The driving operations of the subway system are of great significance in ensuring the safety of trains. There are several hand actions defined in the driving instructions that the driver must strictly execute while operating the train. The actions directly indicate whether equipment is normally operating. Therefore, it is important to automatically sense the region of the driver and detect the actions of the driver from surveillance cameras to determine whether they are carrying out the corresponding actions correctly or not. In this paper, a lightweight two-stage model for subway driver action sensoring and detection is proposed, consisting of a driver detection network to sense the region of the driver and an action recognition network to recognize the category of an action. The driver detection network adopts the pretrained MobileNetV2-SSDLite. The action recognition network employs an improved ShuffleNetV2, which incorporates a spatial enhanced module (SEM), improved shuffle units (ISUs), and shuffle attention modules (SAMs). SEM is used to enhance the feature maps after convolutional downsampling. ISU introduces a new branch to expand the receptive field of the network. SAM enables the model to focus on important channels and key spatial locations. Experimental results show that the proposed model outperforms 3D MobileNetV1, 3D MobileNetV3, SlowFast, SlowOnly, and SE-STAD models. Furthermore, a subway driver action sensoring and detection system based on a surveillance camera is built, which is composed of a video-reading module, main operation module, and result-displaying module. The system can perform action sensoring and detection from surveillance cameras directly. According to the runtime analysis, the system meets the requirements for real-time detection.

## 1. Introduction

With the development of computer technology, researchers apply advanced artificial intelligence technology in the field of transportation, such as traffic sign detection in the field of road traffic [1,2], railway surface [3,4,5,6] and fastener [7,8] defect detection in the field of rail transit. With the rapid development of urban rail transit, the subway system has become the preferred mode of public transportation, which undoubtedly raises higher requirements for the safety of trains. However, there is limited research on subway driver action sensoring and detection based on surveillance cameras. Subway drivers play a crucial role in the safe operation of trains. They need to confirm each step to ensure that no step is missed. The actions of drivers indicate that the current equipment is normally operating. Currently, the monitoring of driver actions is mainly carried out by two surveillance cameras installed in the driver cab. The manual inspection of surveillance videos is used to determine whether the driver has performed the corresponding actions. This method is both inefficient and costly. Therefore, it is of great significance to conduct research on the action sensoring and detection of subway drivers based on surveillance cameras to realize the real-time automatic detection of driver action categories. It can help reduce costs, enhance the operational safety of trains, and improve the intelligence level of urban rail transit monitoring systems.

Subway driver action sensoring and detection (SDASD) belongs to spatial–temporal action detection (STAD), which aims to detect the spatial positions of individuals in the current frame and determine their action categories. In the past, complex handcrafted features have been used, such as spatial–temporal interest points, motion trajectories, etc., for video action recognition [9,10,11,12,13]. These methods achieved good results for simple actions. However, due to the complexity of designing and computing handcrafted features, these methods suffer from slow recognition speed and are not suitable for practical applications.

In recent years, with the rapid development of deep learning, scholars have started to utilize deep neural networks for STAD, which can be categorized into two-stage methods and one-stage methods. The mainstream approach is the two-stage method, where the first stage uses pre-trained object detectors [14,15,16] to generate human region proposals in the current frame. In the second stage, an action recognition network, often utilizing 3D CNNs to extract spatial–temporal features from video clips, is used for action recognition [17]. Gu et al. [18] introduce an AVA dataset and propose a STAD approach. The region proposal network adopts ResNet50, and the action recognition network uses the I3D network [19], which integrates RGB and optical flow, and finally performs action classification. Subsequently, several models are proposed to improve the performance of STAD, such as ACRN (Actor-Centric Relation Network) [20], STEP (Spatio-TEmporal Progressive) [21], LFB (Long-term Feature Banks) [22], SlowFast [23], Context-Aware RCNN [24], ACARN (Actor–Context–Actor Relation Network) [25] and so on. These two-stage models achieve high accuracy, but the human region detection and action recognition are independent, making these models inefficient. In recent studies, several one-stage models are proposed, where a single network is used for both human region detection and action recognition. These models include WOO (Watch Only Once) [26], SE-STAD (Simple and Efficient Spatial–Temporal Action Detector) [27], and DOAD (Decoupled one-stage action detection network) [28]. However, one-stage models face challenges, such as unsatisfied precision. Deep learning-based algorithms have significantly improved the accuracy of STAD, but the deep convolution network models often have a large number of parameters and computation cost, leading to a slow detection speed that does not meet the real-time detection requirements.

To enable deep neural networks to run on devices with limited computational resources, some lightweight networks have been proposed, such as MobileNet [29,30,31] and ShuffleNet [32,33]. ShuffleNetV2 [33] reduces the number of parameters and model size significantly by introducing depthwise separable convolutions. It incorporates channel split and channel shuffle to facilitate information exchange between channels. ShuffleNetV2 [33] strikes a good balance between speed and precision.

As of now, there are no reported studies specifically focusing on SDASD based on surveillance cameras. Some researchers have conducted studies on driver action recognition. For instance, Hu et al. [34] propose the RepC3D model, which combines C3D [35] and RepVGG [36] for recognizing subway driver actions. Suo et al. [37] introduce an improved dense trajectory algorithm for driver action recognition. These studies primarily focus on video-level action recognition, where video clips are used for action classification, without explicitly detecting the region of drivers. Different from the above works, our main objective is to apply advanced artificial intelligence technology in the field of subway driver action detection, which is less relevant research work at present. We propose appropriate improvements on the basis of the existing model to further improve the detection performance. And then based on the improved model, we build a real-time driver action detection system to realize real-time video reading from the surveillance camera to carry out action detection, which makes it possible to deploy the system in the subway cab in the future.

This paper aims to achieve real-time sensing of the region of the subway driver and recognition of their action category based on surveillance cameras. For this purpose, a two-stage model for subway driver action sensoring and detection is proposed. In the first stage, the region of the driver is localized by employing a pre-trained lightweight network called MobileNetV2-SSDLite. The network generates driver candidate region proposals along with confidence scores, which are used for subsequent action recognition. In the second stage, an improved ShuffleNetV2 is proposed to extract the spatial–temporal features of the video clips and recognize the category of actions. To boost the performance of network, a spatial enhanced module is introduced to compensate for spatial information loss caused by downsampling. A new branch with larger convolutional kernels is added to expand the receptive field of the network and a shuffle attention module is used to help the network focus the attention on important channels and spatial positions. Experimental results show that the proposed model outperforms other models, achieving a mAP of 72.44%, 4.87% higher than the baseline. Furthermore, a subway driver action sensoring and detection system based on surveillance cameras is built, which performs real-time action detection directly by reading video from surveillance cameras. It is composed of a video-reading module, main operation module and result-displaying module. The performance of the system shows that it meets the requirements for real-time detection. The main contributions are summarized as follows:

1. A real-time subway driver action sensoring and detection model is proposed, which consists of a driver detection network and an action recognition network. The driver detection network is used to locate the region of the driver in the images, and the action recognition network is employed to recognize the category of the action.
2. A spatial enhanced module is introduced after the first convolution downsampling layer, compensating for the loss of spatial information and enhancing the spatial positions of the feature map. In addition, a dataset specifically for subway driver action sensoring and detection is constructed.
3. A new branch with a large convolutional kernel in the shuffle units is proposed to expand the receptive field, which is crucial for the subsequent action recognition. In addition, the shuffle attention module is introduced to help the network focus the attention on important channels and spatial positions.
4. A real-time subway driver action sensoring and detection system based on surveillance cameras is built, which reads video from surveillance cameras and performs SDASD directly. According to the runtime analysis, the system meets the requirements for real-time detection.

The rest of this paper is organized as follows. Section 2 introduces the problems studied in this paper. In Section 3, a lightweight two-stage model for subway driver action sensoring and detection is proposed. Section 4 introduces the detailed experiments and results. In Section 5, a subway driver action sensoring and detection system is introduced. Section 6 summarizes the conclusions and presents an outlook for future work.

## 2. Problem Statements

Subway drivers play a crucial role in ensuring the safe operation of urban rail transit. They need to confirm each step with their fingers to ensure that no step is missed. The actions of the driver can be used to determine whether the equipment is normally operating or not. Currently, the monitoring of driver actions and their states is primarily performed through the installation of two surveillance cameras in the driver cab (one located at the bottom left corner and the other at the top right corner). The action monitoring uses manual inspection of the recorded videos to check the actions of driver. This manual approach is inefficient and costly.

With the trend of intelligent development in urban rail transit, more and more advanced artificial intelligence algorithms are being applied to detection tasks. However, there is limited research on monitoring subway driver actions. Existing studies mostly focus on classifying driver actions from 2D images, namely recognizing the action category in a single image. Such methods can only identify simple actions without temporal relationships and do not achieve comprehensive action recognition. An action is often composed of multiple consecutive frames with temporal dependencies, and relying on a single image is insufficient for recognizing actions with temporal relationships. Suo et al. [37] study subway driver action recognition by video clips from the perspective of the video level. The actions can be categorized as arrival confirmation, departure confirmation, interval confirmation, platform closing confirmation, and no action. An improved dense trajectory-based method has been proposed for recognizing driver gesture actions. While this method achieves high accuracy, it suffers from slow speed and is used for pre-cropped action video clips; thus, it cannot provide the real-time localization of the driver region and the recognition of driver actions.

**Figure 1:**
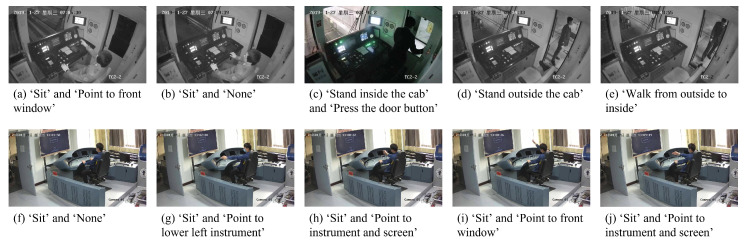
Action categories. The images in the first rows are from the surveillance video of the driver cab, and the images in the second rows are from simulated video.

In this paper, the action sensoring and detection of subway drivers based on surveillance cameras is studied, aiming to sense the region of the driver and recognize their current actions from surveillance videos. According to surveillance videos in subway driver cabs, driver actions and states are categorized into 11 classes as shown in Figure 1, including sitting (Sit), standing inside the cab (StinCab), standing outside the cab (StoutCab), walking from inside to outside (WafrI2O), walking from outside to inside (WafrO2I), pointing to the instrument and screen (Po2InSc), pointing to the front window (Po2FrWin), pointing to the lower left instrument (Po2LLin), pressing the door button (PrDoBu), pushing the instrument (PuIn), and no action (None). For the task of driver region localization, the lightweight object detection algorithm MobileNetv2-SSDLite is employed to locate the region of the driver in the image, and the coordinates and confidence scores are obtained. These coordinates and scores are used as regions of interest (ROIs) and fed to the action recognition network. For driver action recognition, an improved lightweight ShuffleNetv2 is proposed to extract spatial–temporal features from multiple frames of input. The ROIs from the driver region localization task are mapped onto the final feature map, followed by ROI pooling to generate fixed-size features for ROIs, and then processed through fully connected layers for action recognition. To evaluate the real-time performance of the proposed model, a subway driver action sensoring and detection system based on surveillance cameras is built, consisting of three parts: video-reading module, main operation module, and result-displaying module. The video-reading module stores the video streams in a reading queue, the main operation module samples frames from the queue and performs driver region localization and driver action recognition on the sampled frames, and the result-displaying module renders the results on the frames and composes the video for display on the screen. A surveillance camera is installed, and the system can perform action sensoring and detection on the video streams from the surveillance camera. According to the runtime of each module, the system achieves real-time detection.

## 3. Methodology

### 3.1. Overall Framework

**Figure 2:**
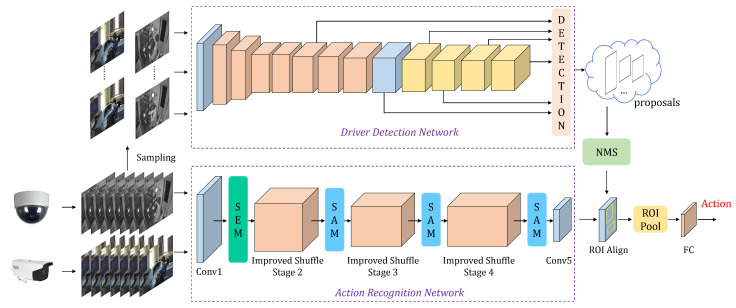
The overall framework of the model.

The overall framework of the model is shown in Figure 2. The model is composed of the driver detection network and the action recognition network. The driver detection network generates proposals of the driver region, and the action recognition network recognizes the current action category of the driver. To achieve fast and accurate detection, lightweight networks are employed for both tasks. For the driver detection network, a pre-trained MobileNetV2-SSDLite [30] is utilized, which has a small number of parameters and model size, allowing it to quickly and accurately detect the region of the driver. For the action recognition network, an improved ShuffleNetV2 is proposed. The network also has a small number of parameters and model size, enabling the fast and accurate recognition of actions.

### 3.2. Driver Detection Network

In this paper, the pre-trained MobileNetv2-SSDLite is adopted for the driver detection network. It aims to sense and locate the region of the driver in the image and obtain the coordinates and confidence scores. The SSD [16] is a classic one-stage object detection algorithm that can simultaneously perform object localization and classification in a single stage. It combines the advantages of the anchor-based mechanism from region proposal algorithms and the regression-based algorithm in one-stage methods, resulting in high accuracy and fast detection speed. The original SSD uses VGG16 as a base network. However, the large number of parameters in the VGG16 makes it unsuitable for running on resource-limited embedded devices and mobile devices. To address this issue, the SSDLite object detection network based on MobileNetV2 is proposed, which reduces the number of parameters and computation. Specifically, the VGG16 is replaced with MobileNetV2 for feature extraction. Additionally, instead of using regular convolutions, the extra convolution layers in SSDLite utilize depthwise separable convolutions (DWConv) as the basic structure. The input size of image is 320 × 320. Predictions are made by using six different-sized feature maps when detecting, and proposals of the driver region along with confidence scores are obtained. These proposals are then selected by non-maximum suppression (NMS) to obtain the final proposals, used for subsequent action recognition.

### 3.3. Action Recognition Network

#### 3.3.1. ShuffleNetV2

**Figure 3:**
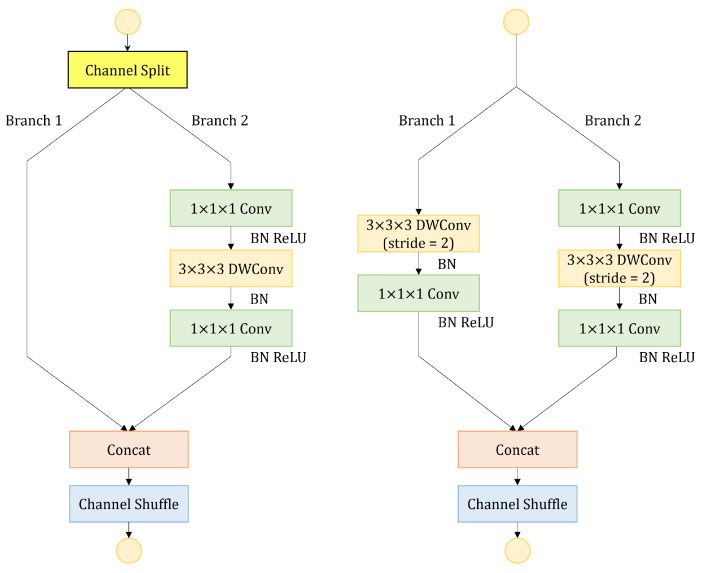
The structure of the shuffle units.

**Figure 4:**
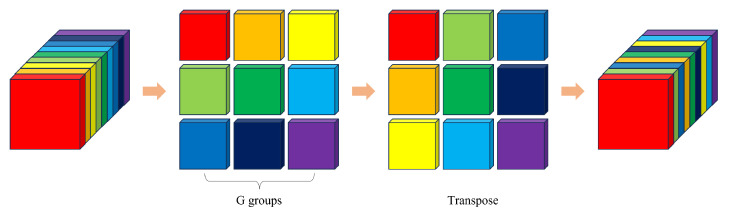
The structure of the channel shuffle.

Three-dimensional ShuffleNetV2 [33] is a lightweight network with small number of parameters and computation. The shuffle units of 3D ShuffleNetV2 are shown in Figure 3. When the stride is one, the channels are split into two branches. Branch 1 does not have any operations, while branch 2 adopts DWConv. The outputs of branch 1 and branch 2 are concatenated, followed by a channel shuffle module. When the stride is two, the input is processed by two branches. The outputs of branch 1 and branch 2 are concatenated. The channel shuffle operation allows for information exchange between channels. The channel shuffle module is shown in Figure 4. For a given feature map with a specific number of channels, the channels are first divided into G groups. Then, the groups are transposed and rearranged to obtain the shuffled feature map. The channel shuffle operation does not introduce any parameters and achieves channel-wise information exchange through simple grouping and transposition operations.

#### 3.3.2. Spatial Enhanced Module (SEM)

**Figure 5:**
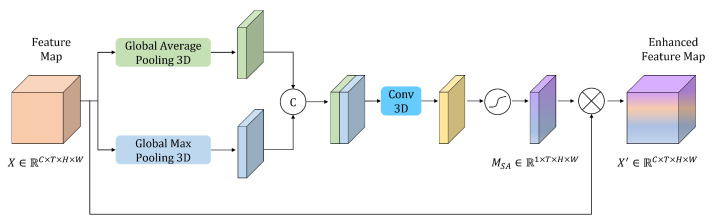
The structure of the spatial enhanced module.

When the network performs convolutional downsampling, the size of the feature maps decreases. Though this allows for capturing high-level features, downsampling leads to spatial information loss. To reduce the information loss, a SEM is added after the first convolution to enhance the spatial representation capability of network. The specific structure is shown in Figure 5. Firstly, the global average pooling and global max pooling are adopted. The resulting feature maps are then concatenated and processed by a 3D convolution to extract features. An activation function is applied to enhance the representation ability of the network, and the feature maps are multiplied element-wise with the original feature maps to obtain the enhanced feature maps. The formulas are denoted as follows:

(1) $$M_{SA}=\sigma \left(conv\left(\left[AvgPool\left(X\right);MaxPool\left(X\right)\right]\right)\right)$$

(2) $$X^{{}^{\prime }}=X⊗M_{SA}$$

#### 3.3.3. Improved Shuffle Units (ISUs)

**Figure 6:**
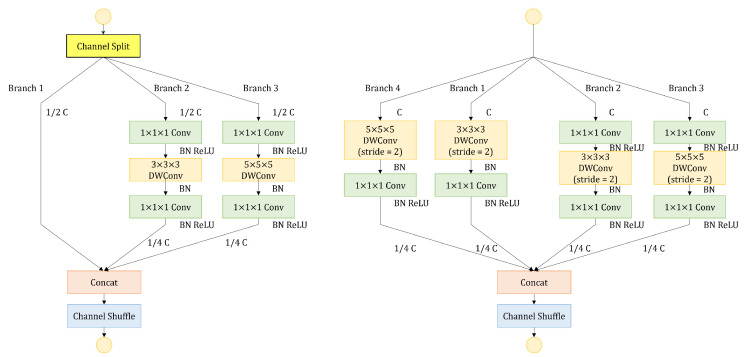
The structure of the improved shuffle units.

The receptive field of the network can be expanded by using large convolution kernels, which is crucial for subsequent tasks. Inspired by it, a new branch with a 5 × 5 × 5 kernel size is added to the ShuffleNet units as shown in Figure 6. When the stride is 1, branch 3 is added to obtain a larger receptive field. The rest of branch 3 is the same as branch 2. To ensure that the final concatenation has the same number of channels, the output channels of the last convolution in branch 2 and branch 3 are set to 1/4 of the input channels. Branch 1, branch 2, and branch 3 are then concatenated to obtain feature maps, followed by a channel shuffle module. When the stride is 2, branch 3 and branch 4 are added with a bigger kernel size. The output channels of the last convolution in all branches are set to 1/4 of the input channels. Branch 1, branch 2, branch 3, and branch 4 are then concatenated to obtain the final feature maps, followed by a channel shuffle module. The introduction of improved shuffle units increases the number of computations, about 1.5 times to 2 times as much as the original shuffle units. The detailed calculation procedure is shown in Appendix A.

#### 3.3.4. Shuffle Attention Module (SAM)

**Figure 7:**
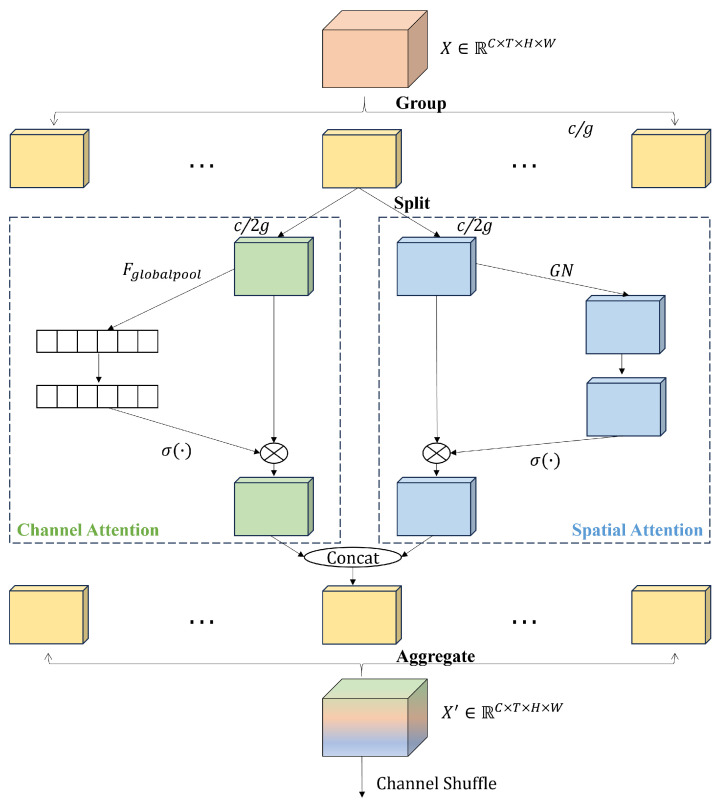
The structure of the shuffle attention module.

The attention module allows the network to focus on important features and suppress unimportant features. There are two main types of attention mechanisms: channel attention and spatial attention. Channel attention focuses on “what”, while spatial attention focuses on “where”. In this paper, to boost the representation ability in both the spatial and channel dimensions of the feature maps, the shuffle attention module (SAM) [38] is added after each improved shuffle stage. The structure of SAM is shown in Figure 7. First, the feature maps are divided into *g* groups along the channel dimension. Each group is further divided into two branches, namely, the channel attention branch and the spatial attention branch. These branches are responsible for generating different channel and spatial importance weights.

Channel attention branch: Instead of using the traditional SE [39] module, which introduces a large number of parameters, a simple combination of global average pooling, scale, and sigmoid is adopted. Firstly, global average pooling is used to embed global information, generating $s\in R^{\frac{c}{2g}\times 1\times 1\times 1}$.

(3) $$s=F_{gp}\left(X_{k1}\right)=\frac{1}{T\times H\times W}\sum_{i=1}^{T}\sum_{j=1}^{H}\sum_{k=1}^{W}X_{k1}\left(i,j,k\right)$$

(4) $$X_{k1}^{{}^{\prime }}=\sigma \left(F_{c}\left(s\right)\right)\cdot X_{k1}=\sigma \left(W_{1}s+b_{1}\right)\cdot X_{k1}$$

Spatial attention branch: Unlike the channel attention branch, spatial attention focuses on the spatial dimension. Firstly, Group Norm (GN) is used to obtain statistical information along the spatial dimensions. Then, Fc(.) is applied to enhance the spatial attention. The formula is denoted as follows:

(5) $$X_{k2}^{{}^{\prime }}=\sigma \left(W_{2}\cdot GN\left(X_{k2}\right)+b_{2}\right)\cdot X_{k2}$$

Aggregation: After obtaining the channel attention weights and spatial attention weights, it is necessary to aggregate them. Firstly, a simple concatenation operation is adopted. Then, inter-group information exchange is performed by channel shuffle module.

The computation of the grouping operation in SAM is about $\frac{1}{4g^{2}}$ of that of the non-grouping operation. The detailed calculation procedure is shown in Appendix B.

#### 3.3.5. Network Structure

After a detailed introduction of each component of the network, the complete action recognition network is presented along with its structure and specific parameters in Table 1. The network consists of two standard convolutions, one max pooling, one global average pooling, one SEM module, three improved shuffle stages, three SAM modules, one ROI Align and Pooling, and one fully connected layer. In Table 1, T represents the number of input frames, and there is no downsampling in the temporal dimension.

## 4. Experiments

### 4.1. Dataset Preparation

The videos of the driver cab used in the experiment are from Beijing Metro Line 9, with a video resolution of 1280 × 720. The simulation video is recorded by the surveillance camera installed in the lab, with a video resolution of 1920 × 1080. Each raw surveillance video is about an hour long, and if it is fed directly into the model, it requires a huge amount of memory and computing resources. Therefore, in order to better sense and detect the category of the subway driver action, 328 video clips are cropped with a duration of 10 s from the original driver cab videos and simulation videos, of which 163 clips are from the actual surveillance video and 165 clips are from laboratory simulation videos. Each clip contains two action labels. The action labels includes sitting (Sit), standing inside the cab (StinCab), standing outside the cab (StoutCab), walking from inside to outside (WafrI2O), walking from outside to inside (WafrO2I), pointing to the instrument and screen (Po2InSc), pointing to the front window (Po2FrWin), pointing to the lower left instrument (Po2LLin), pressing the door button (PrDoBu), pushing the instrument (PuIn), and no action (None). The number of labeled actions is shown in Table 2. The driver detection network does not require additional datasets. As the proposed model works, the frames for the driver detection network are sampled from the input video clip and no additional datasets are required.

**Figure 8:**
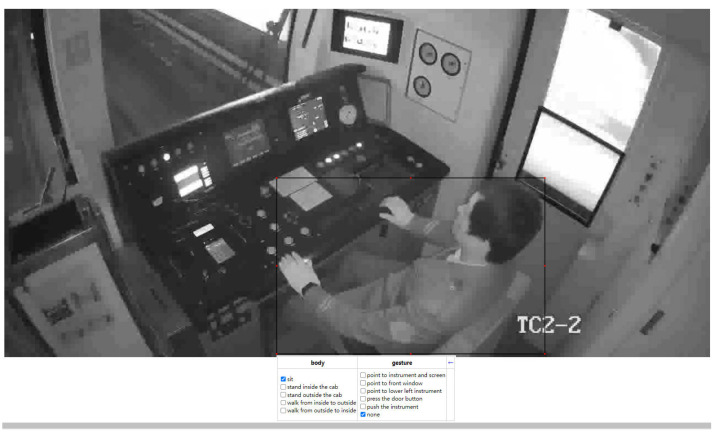
The annotation process.

The annotation method refers to the AVA dataset format [18]. First, the video is extracted into a series of frames with an FPS of 30. The AVA dataset format does not label all frames but annotates 1 frame per second. Therefore, in the spatial–temporal detection dataset of the subway driver action, the first frame per second is annotated [40]. Since the first and last 2 s of the videos are not involved in detecting, only images with indexes of 61, 91, 121, 151, 181, 211, 241 are labeled for each video clip with a duration of 10 s. In order to quickly label the region of the driver, the pre-trained YOLOv5 [41] is used to detect the region of the driver, and the coordinates and confidence scores of the proposals are obtained as rough labeling, and then the rough labeling is imported into the VIA [42] labeling tool for action category labeling. The annotation process is shown in Figure 8.

The detailed process by which MobileNetV2-SSDLite results are used by improved shuffleNetV2 to drive the action classification task is as follows.

Taking a cropped video clip for example, the first and last 2 s of the videos are not involved in detecting (AVA dataset format), and the index of 61, 91, 121, 151, 181, 211, and 241 frames are labeled. Therefore, taking these seven frames as the center, and sampling eight frames each, we can obtain seven clips for the model training, where each clip has eight frames.

Taking the frame with index 61 as an example, with 61 as the center and an interval of 8, a total of 8 frames are sampled, that is, the index corresponding to the sampled frames is (29, 37, 45, 53, 61, 69, 77, 85), and the action label is the same as the label of the frame with index 61. For the driver detection network, these eight frames are input in sequence to obtain the driver proposals (namely anchors) in the eight frames. Then, the non-maximum suppression (NMS) is then used to filter invalid anchors that exceed a fixed threshold. The retained anchors after NMS are then mapped on the feature map of the last convolution layer of the improved ShuffleNetV2 (namely the output feature map of conv5, as shown in Figure 1). For the action recognition network, these eight frames are taken as an input. The region of the driver on the feature map, namely the region of interest (ROI), can be obtained, and then the ROIs which have different sizes are transformed into fixed sizes by ROI pooling, and finally the fixed size features are sent to the fully connected layer for action classification.

### 4.2. Evaluation Indicators and Experimental Details

The purpose of subway driver action sensoring and detection is to sense and locate the region of the driver and recognize the category of the action, paying more attention to driver action recognition. A common evaluation indicator is mAP (mean Average Precision), which is the average of AP in all action categories.

In this paper, all the models are implemented by PyTorch and trained on 1 NVIDIA A6000 GPU. The CPU is Inter(R) Xeon(R) silver 4314 @2.4 GHz.

The input of the model is eight frames, which are sampled at equal intervals. The interval in this paper is set to eight, that is, one frame is sampled every eight frames. When model training, eight images are scaled to 256 × 256 after sampling, and horizontal flip is introduced to augment the dataset. For model testing, the height of the image is scaled to 256, and the width is scaled proportionally. The parameters are set as follows: the optimizer is SGD, the initial learning rate is 0.01, the weight momentum is 0.00003, and the learning rate decay strategy is cosine annealing, where warmup_iters is 500, warmup_ratio is 0.1, the minimum learning rate is 0.00001, and the training epoch is 200.

### 4.3. Experimental Results

For the driver detection network, the Intersection over Union (IoU) threshold is set to 0.5. For the action recognition network, the prediction score threshold is set to 0.9. The experimental results are shown in Table 3. The baseline is the original 3D ShuffleNetV2 [33]. As can be seen from Table 3, SEM, ISU, and SAM can improve the performance of the model. ISU brings the most obvious gain; the mAP can increase 4.13%, and the number of parameters is smaller than the baseline. When all modules are added to the network, the mAP increases to 72.44%, 4.87% higher than the baseline.

**Figure 9:**
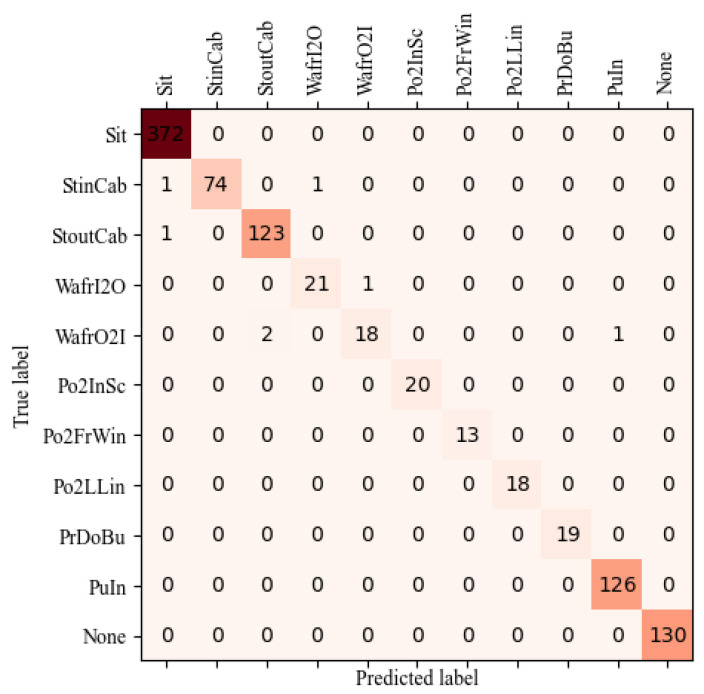
The confusion matrix of improved ShuffleNetV2.

Table 4 shows the AP of each action. It can be seen that the introduction of SEM, ISU and SAM improve the AP of most action categories, among which walking from inside to outside has the most obvious improvement, from 0.5143 to 0.8309, improved by 31.66%, followed by pressing the door button, which increases from 0.2172 to 0.3276, an improvement of 11.04%. Though the AP of walking from outside to inside, pointing to the instrument and screen, and pushing the instrument decrease slightly, other categories of actions have different amplitudes of increment. It can be concluded that the introduction of SEM, ISU and SAM improve the performance of the model. In addition, it is seen that the AP of pressing the door button action is low, owing to the small number of action instances in the actual surveillance video, and this action is not simulated due to the constraints of the operation console, which further leads to a smaller number of actions than other actions, resulting in the model not learning the feature of action well. Figure 9 shows the confusion matrix for the recognition results of the proposed model. The action recognition network can almost accurately classify all kinds of actions; only seven actions are classified incorrectly. Of these seven actions, one StinCab and one StoutCab are classified as Sit because the driver is close to the seat in the two video clips, leading to the wrong classification. One StinCab is classified as WafrI2O because the StinCab occurs at the junction where the two actions occur, resulting in a classification error. Two WafrO2I are classified as StoutCab because these actions are both outside the cab and the action after StoutCab is WafrO2I. One WafrI2O is classified as WafrO2I because at the door, WafrI2O is similar to WafrO2I. One WafrO2I is classified as PuIn because in the real cab video, the action behind WaFrO2I is PuIn, resulting in a recognition error at the action connection. Overall, it can be seen that the proposed model can well recognize the action categories of the driver.

In order to prove that the SAM module is better than the SE module, we carried out a comparative experiment. Table 5 shows the comparison results. It can see that the SAM has fewer parameters and better performance than the SE module.

In order to evaluate the performance of the proposed network, 3D MobileNetV1 [29], 3D MobileNetV3 [31], SlowFast-R50 [23], SlowOnly-R50 [23], and SE-STAD [27] are selected for comparison. The experimental results are shown in Table 6. The results show that the proposed network has a higher mAP and a smaller number of parameters and model size than the compared networks. Table 7 shows the AP of each action. It can be seen that the APs of the proposed model are better than the compared models in most action categories, and the proposed network reaches the state of the art.

**Figure 10:**
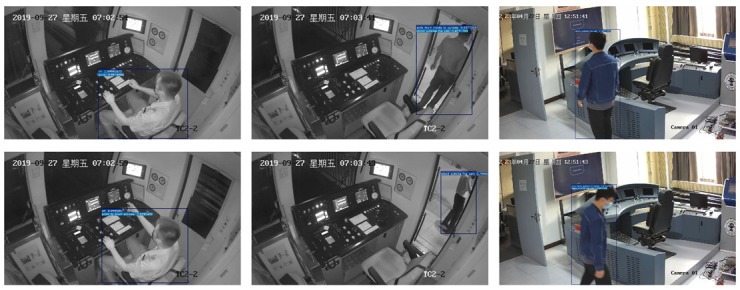

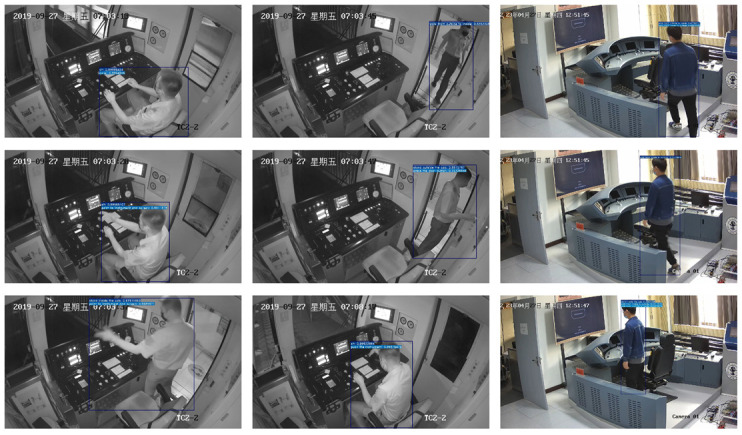
The action sensoring and detection result of the subway driver.

Figure 10 shows the driver action detection results from the actual surveillance video in the driver cab. It can be seen that the model can precisely locate the region of the driver and recognize the category of action.

## 5. Subway Driver Action Sensoring and Detection System

### 5.1. System Structure

**Figure 11:**
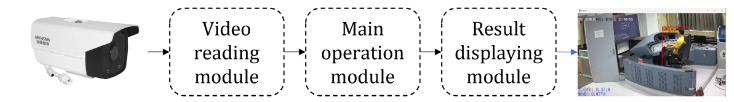
The structure of subway driver action sensoring and detection system.

Based on the proposed model, a subway driver action sensoring and detection system (SDASD) is built, aiming to read the video from the surveillance camera in real-time and conduct SDASD directly. The system structure is shown in Figure 11. The system is composed of a video-reading module, main operation module and result-displaying module.

#### 5.1.1. Video-Reading Module

The real-time video is read from the surveillance camera, and video frames are stored in the reading queue for the main operation module.

#### 5.1.2. Main Operation Module

Frames are sampled from the reading queue according to the given sampling strategy (that is, sampling 1 frame at an interval of 8, and sampling 8 frames in total). The eight frames are sent to the driver detection network to predict the region of the driver, and the coordinates and confidence scores of the proposals are obtained. The region proposals are drawn in the frames, which are taken as the ROIs in the subsequent action recognition. In addition, the eight frames are pre-processed for the action recognition network. In the last layer of convolution, the ROIs in the driver detection network are mapped on the feature map, and finally the ROI pooling is carried out to obtain the fixed-size feature map. Finally, the action categories and corresponding confidence scores are predicted through the fully connected layer.

#### 5.1.3. Result-Displaying Module

The result-displaying module mainly draws the action recognition results on the frames, including the action category and its corresponding confidence score, then synthesizes the video at a fixed frame rate, and finally displays it on the screen.

### 5.2. Performance Evaluation

**Figure 12:**
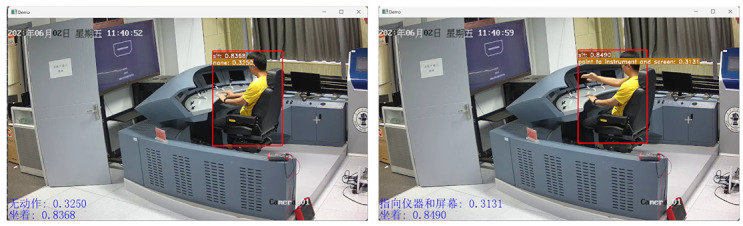
The results of real-time detection system.

To evaluate the performance of the system, experiments are conducted on a personal laptop with a Core (TM) i5-12500H 2.5 GHz CPU and an NVIDIA RTX 2050 (4 GB) GPU. Since the input images are sampled from 64 frames (about 64/30 = 2.13 s), that is, the model needs to complete the whole process (video-reading module, main operation module, and result-displaying module) within 2.13 s to meet the real-time detection requirements. Table 8 shows the time consumed of each module. It can be seen that the complete runtime of our model is between 0.6 s and 0.75 s, which is much less than 2.13 s. Compared with the other models, our model is better regarding total runtime. Therefore, it is concluded that the model meets the requirements of real-time action detection from surveillance cameras. The results of the real-time detection system are shown in Figure 12.

## 6. Conclusions

In this paper, a lightweight two-stage model for subway driver action sensoring and detection based on surveillance cameras is proposed. It consists of the driver detection network and the action recognition network. The driver detection network adopts MobileNetV2-SSDLite, with the purpose of locating the region of the driver. The action recognition network employs the improved ShuffleNetV2 to extract spatial–temporal features and recognizes the category of action. The proposed network has a smaller number of parameters and model size than the compared networks. The experimental results show that the proposed network outperforms the compared networks, with a mAP of 72.44%, 4.87% higher than the baseline. Then a subway driver action sensoring and detection system is built based on the proposed model to realize real-time detection from surveillance cameras. The system runs on a personal laptop; according to the runtime of the system, it takes 0.6 to 0.75 s for a whole process, which is less than the video duration of 2.13 s. It can be seen that the system meets the real-time detection requirements.

In the future research, we will further optimize our system, expand the dataset, and improve the performance. In addition, the action statistics function is taken into account for the subway driver action sensoring and detection to count the number of actions completed by the driver.

## Figures and Tables

**Table 1:** Structure and parameters of the action recognition network.

Operator	Output Size	KSize	Stride	Rep.	Output Channels
Frames	T × 256 × 256	-	-	-	3
Conv1	T × 128 × 128	3 × 3 × 3	(1,2,2)	1	24
MaxPool	T × 64 × 64	3 × 3 × 3	(1,2,2)	1	24
SEM	T × 64 × 64	-	-	-	24
Improved Shuffle Stage2	T × 32 × 32	-	(1,2,2), (1,1,1)	1, 3	116
SAM	T × 32 × 32	-	-	-	116
Improved Shuffle Stage3	T × 16 × 16	-	(1,2,2), (1,1,1)	1, 7	232
SAM	T × 16 × 16	-	-	-	232
Improved Shuffle Stage4	T × 16 × 16	-	(1,1,1)	4	464
SAM	T × 16 × 16	-	-	-	464
Conv5	T × 16 × 16	1 × 1 × 1	(1,1,1)	1	1024
ROI Align & Pool	1 × 8 × 8	-	-	-	1024
Global Average Pool	1 × 1 × 1	-	-	-	1024
Fully Connected	-	-	-	-	12

KSize represents kernel size, Rep. represents repeat number.

**Table 2:** The number of labeled actions.

Action	Training Set	Testing Set	Total
Sit	953	402	1355
StinCab	157	89	246
StoutCab	326	147	473
WafrI2O	82	25	107
WafrO2I	91	23	114
Po2InSc	109	31	140
Po2FrWin	62	21	83
Po2LLin	61	22	83
PrDoBu	98	58	156
PuIn	268	150	418
None	465	181	646
**Total**	2672	1149	3821

**Table 3:** Ablation experiments.

Baseline	SEM	ISU	SAM	mAP
**✓**				0.6757
**✓**	**✓**			0.6842
**✓**		**✓**		0.7170
**✓**			**✓**	0.7114
**✓**	**✓**	**✓**	**✓**	**0.7244**

**Table 4:** The AP of actions.

Action	Baseline	+SEM	+ISU	+SAM	+SEM+ISU+SAM
Sit	0.8760	0.8672	0.8804	0.8680	**0.8817**
StinCab	0.7865	0.8090	0.8315	0.8090	**0.8315**
StoutCab	0.8231	**0.8503**	0.8360	0.8298	0.8296
WafrI2O	0.5143	0.8696	**0.8783**	0.8200	0.8309
WafrO2I	0.8239	0.7826	**0.8261**	0.7712	0.7666
Po2InSc	0.5839	0.4073	**0.6144**	0.4315	0.5823
Po2FrWin	0.5714	0.6190	0.5159	0.6667	**0.6122**
Po2LLin	0.7727	0.7246	0.7702	0.7727	**0.8182**
PrDoBu	0.2172	0.1724	0.2165	0.3575	**0.3276**
PuIn	0.7967	0.7463	**0.8240**	0.8047	0.7865
None	0.6670	0.6774	0.6936	0.6940	**0.7013**
mAP	0.6757	0.6842	0.7170	0.7114	**0.7244** (+4.87%)

**Table 5:** Comparison between SE and SAM.

Model	Model Size	Parameters	GFlops	mAP
+SEM+ISU+SE	10.50 M	1,329,416	6.36	0.7230
+SEM+ISU+SAM	10.26 M	1,294,740	6.36	0.7244

**Table 6:** Comparison with other networks.

Model	mAP	Parameters	Model Size	GFlops
3D MobileNetV1 [29]	0.6164	3,310,284	25.43 M	14.60
3D MobileNetV3 [31]	0.7189	1,165,964	9.07 M	3.39
SlowFast-R50 [23]	0.6095	33,671,220	257.46 M	193.57
SlowOnly-R50 [23]	0.6630	31,659,084	241.91 M	166.08
SE-STAD [27]	0.7068	40,650,557	310 M	213.65
**ours**	**0.7244**	1,294,740	10.26 M	6.36

**Table 7:** The AP of actions.

Action	3D MobileNetV1 [29]	3D MobileNetV3 [31]	SlowFast-R50 [23]	SlowOnly-R50 [23]	SE-STAD [27]	Ours
Sit	0.8742	0.8737	0.8753	0.869	0.9801	0.8817
StinCab	0.7978	0.8427	0.7978	0.8539	0.8202	0.8315
StoutCab	0.8298	0.8299	0.8639	0.8502	0.9048	0.8296
WafrI2O	0.76	0.8345	0.64	0.781	0.4	0.8309
WafrO2I	0.5652	0.7826	0.3913	0.6957	0.3478	0.7666
Po2InSc	0.4649	0.5755	0.5066	0.5145	0.7041	0.5823
Po2FrWin	0.1905	0.6444	0.4459	0.4238	0.7963	0.6122
Po2LLin	0.7273	0.7727	0.5455	0.6364	0.8636	0.8182
PrDoBu	0.1552	0.1551	0.1207	0.1207	0.1034	0.3276
PuIn	0.7929	0.8174	0.8266	0.8259	0.9267	0.7865
None	0.6231	0.7789	0.6906	0.7222	0.9282	0.7013
mAP	0.6164	0.7189	0.6095	0.663	0.7068	0.7244

**Table 8:** The runtime of each module.

Model	Video-Reading Module	Main Operation Module	Result-Displaying Module	Total
3D MobileNetV1 [29]	280–350 ms	120–180 ms	300–350 ms	700–880 ms
3D MobileNetV3 [31]	250–350 ms	100–180 ms	280–350 ms	630–880 ms
SlowFast-R50 [23]	280–350 ms	360–450 ms	380–450 ms	1020–1250 ms
SlowOnly-R50 [23]	280–350 ms	220–280 ms	250–300 ms	750–930 ms
ours	250–300 ms	100–150 ms	250–300 ms	600–750 ms

**Figure A1:**
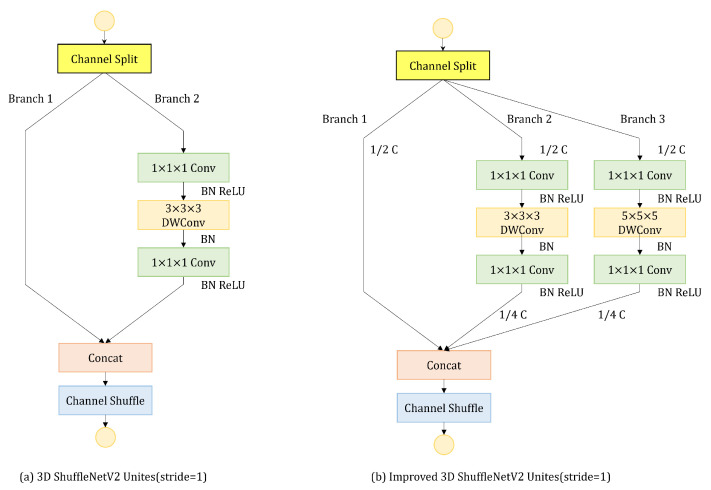
The structures of the original shuffle units and improved shuffle units (stride = 1).

**Figure A2:**
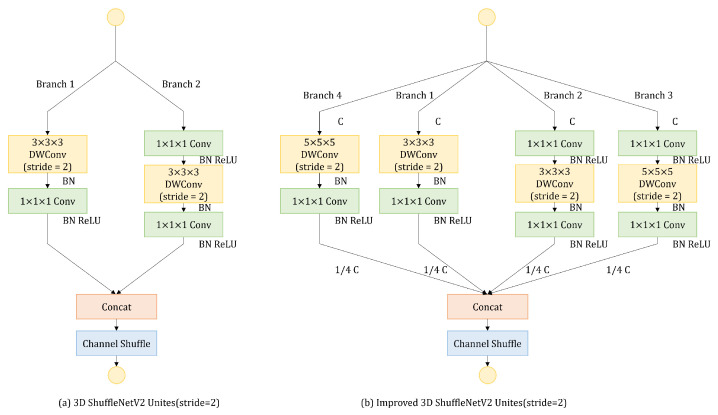
The structures of original shuffle units and improved shuffle units (stride = 2).

## Data Availability

Data are contained within the article.

## Appendix A

The additional computation resulting from the improved shuffle unit (ISU) is calculated as follows.

When the stride is one, the input shape is $X\in R^{C\times T\times H\times W}$, and the output shape is $X^{{}^{\prime }}\in R^{C\times T\times H\times W}$. The structures of the original shuffle units and improved shuffle units are shown in Figure A1.

The computation of 3D ShuffleNetV2 (stride = 1):

(A1) $$\begin{array}{cc} Comput\_original1 & =\left(2\times \frac{C}{2}\times 1\times 1\times 1-1\right)\times \frac{C}{2}\times T\times H\times W\\ \; & +\left(\frac{2\times \frac{C}{2}\times 3\times 3\times 3}{\frac{C}{2}}-1\right)\times \frac{C}{2}\times T\times H\times W\\ \; & +\left(2\times \frac{C}{2}\times 1\times 1\times 1-1\right)\times \frac{C}{2}\times T\times H\times W\\ \; & =\frac{C}{2}\times T\times H\times W\times \left(2C+51\right) \end{array}$$

The computation of improved 3D ShuffleNetV2 (stride = 1):

(A2) $$\begin{array}{cc} Comput\_improved1 & =\left(2\times \frac{C}{2}\times 1\times 1\times 1-1\right)\times \frac{C}{2}\times T\times H\times W\\ \; & +\left(\frac{2\times \frac{C}{2}\times 3\times 3\times 3}{\frac{C}{2}}-1\right)\times \frac{C}{2}\times T\times H\times W\\ \; & +\left(2\times \frac{C}{2}\times 1\times 1\times 1-1\right)\times \frac{C}{4}\times T\times H\times W\\ \; & +\left(2\times \frac{C}{2}\times 1\times 1\times 1-1\right)\times \frac{C}{2}\times T\times H\times W\\ \; & +\left(\frac{2\times \frac{C}{2}\times 5\times 5\times 5}{\frac{C}{2}}-1\right)\times \frac{C}{2}\times T\times H\times W\\ \; & +\left(2\times \frac{C}{2}\times 1\times 1\times 1-1\right)\times \frac{C}{4}\times T\times H\times W\\ \; & =\frac{C}{2}\times T\times H\times W\times \left(3C+299\right) \end{array}$$

It can be seen that when the stride is one, the introduction of improved shuffle units increases the number of computations, about 1.5 times as much.

When the stride is two, the input shape is $X\in R^{C\times T\times H\times W}$, and the output shape is $X^{{}^{\prime }}\in R^{C\times T\times \frac{H}{2}\times \frac{W}{2}}$. The structures of the original shuffle units and improved shuffle units are shown in Figure A2.

The computation of 3D ShuffleNetV2 (stride = 2):

(A3) $$\begin{array}{cc} Comput\_original2 & =(2\times C\times 1\times 1\times 1-1)\times C\times T\times H\times W\\ \; & +2\times \left(\frac{2\times C\times 3\times 3\times 3}{C}-1\right)\times C\times T\times \frac{H}{2}\times \frac{W}{2}\\ \; & +2\times \left(2\times C\times 1\times 1\times 1-1\right)\times \frac{C}{2}\times T\times \frac{H}{2}\times \frac{W}{2}\\ \; & =C\times T\times \frac{H}{2}\times \frac{W}{2}\times \left(10C+101\right) \end{array}$$

The computation of improved 3D ShuffleNetV2 (stride = 2):

(A4) $$\begin{array}{cc} Comput\_improved2 & =2\times (2\times C\times 1\times 1\times 1-1)\times C\times T\times H\times W\\ \; & +2\times \left(\frac{2\times C\times 3\times 3\times 3}{C}-1\right)\times C\times T\times \frac{H}{2}\times \frac{W}{2}\\ \; & +2\times \left(\frac{2\times C\times 5\times 5\times 5}{C}-1\right)\times C\times T\times \frac{H}{2}\times \frac{W}{2}\\ \; & +4\times \left(2\times C\times 1\times 1\times 1-1\right)\times \frac{C}{4}\times T\times \frac{H}{2}\times \frac{W}{2}\\ \; & =C\times T\times \frac{H}{2}\times \frac{W}{2}\times \left(18C+595\right) \end{array}$$

It can be seen that the introduction of four branches increases the number of computations, about twice as much.

## Appendix B

The computation of the grouping operation and non-grouping operation is calculated as follows.

The computation of the grouping operation: (*g* groups)

(1) The computation of channel attention:

(A5) $$\left(2\times \frac{C}{2g}\times 1\times 1\times 1-1\right)\times \frac{C}{2g}\times T\times H\times W$$

(2) The computation of spatial attention:

(A6) $$\left(2\times \frac{C}{2g}\times 1\times 1\times 1-1\right)\times \frac{C}{2g}\times T\times H\times W$$

(3) The total computation of grouping operation:

(A7) $$2\times \left(2\times \frac{C}{2g}\times 1\times 1\times 1-1\right)\times \frac{C}{2g}\times T\times H\times W$$

The computation of the non-grouping operation:

(1) The computation of channel attention:

(A8) (2×C×1×1×1−1)×C×T×H×W

(2) The computation of spatial attention:

(A9) (2×C×1×1×1−1)×C×T×H×W

(3) The total computation of non-grouping operation:

(A10) 2×(2×C×1×1×1−1)×C×T×H×W

The computation ratio of grouping and non-grouping operation:

(A11) $$\frac{group}{non\_group}=\frac{2\times \left(2\times \frac{C}{2g}\times 1\times 1\times 1-1\right)\times \frac{C}{2g}\times T\times H\times W}{2\times (2\times C\times 1\times 1\times 1-1)\times C\times T\times H\times W}\approx \frac{1}{4g^{2}}$$

It can see that the grouping operation can reduce the number of computations. The computation of grouping operation is about $\frac{1}{4g^{2}}$ of that of the non-grouping operation.
